# Achieving Tiered Model Quality in 3D Structure from Motion Models Using a Multi-Scale View-Planning Algorithm for Automated Targeted Inspection

**DOI:** 10.3390/s19122703

**Published:** 2019-06-16

**Authors:** Trent J. Okeson, Benjamin J. Barrett, Samuel Arce, Cory A. Vernon, Kevin W. Franke, John D. Hedengren

**Affiliations:** 1 Department of Chemical Engineering, Ira A. Fulton College of Engineering and Technology, Brigham Young University, 350 Clyde Building, Provo, UT 84602, USA; okesontj@byu.net (T.J.O.); samuelarce@byu.edu (S.A.); cvernon3@byu.edu (C.A.V.); 2 Department of Civil and Environmental Engineering, Ira A. Fulton College of Engineering and Technology, Brigham Young University, 368 Clyde Building, Provo, UT 84602, USA; benjamin.barrett@aecom.com (B.J.B.); kevin_franke@byu.edu (K.W.F.)

**Keywords:** structure from motion, unmanned aerial vehicles, dam inspection, automated inspection, Multi-Scale, view-planning

## Abstract

This study presents a novel multi-scale view-planning algorithm for automated targeted inspection using unmanned aircraft systems (UAS). In industrial inspection, it is important to collect the most relevant data to keep processing demands, both human and computational, to a minimum. This study investigates the viability of automated targeted multi-scale image acquisition for Structure from Motion (SfM)-based infrastructure modeling. A traditional view-planning approach for SfM is extended to a multi-scale approach, planning for targeted regions of high, medium, and low priority. The unmanned aerial vehicle (UAV) can traverse the entire aerial space and facilitates collection of an optimized set of views, both close to and far away from areas of interest. The test case for field validation is the Tibble Fork Dam in Utah. Using the targeted multi-scale flight planning, a UAV automatically flies a tiered inspection using less than 25% of the number of photos needed to model the entire dam at high-priority level. This results in approximately 75% reduced flight time and model processing load, while still maintaining high model accuracy where needed. Models display stepped improvement in visual clarity and SfM reconstruction integrity by priority level, with the higher priority regions more accurately modeling smaller and finer features. A resolution map of the final tiered model is included. While this study focuses on multi-scale view planning for optical sensors, the methods potentially extend to other remote sensors, such as aerial LiDAR.

## 1. Introduction

Infrastructure monitoring is a significant challenge for businesses and government agencies. With aging equipment and infrastructure, regular monitoring is important to ensure the safety of existing structures; however, limited budgets prevent more frequent monitoring. Recently there has been significant interest in using unmanned aerial vehicles (UAVs) to perform infrastructure monitoring inspections [1,2]. UAVs have grown in popularity due to high versatility, allowing for a wide variety of applications. UAVs can enter areas that are dangerous for people, perform routine surveillance, and complete difficult inspection tasks. Auxiliary sensors, including multi-spectral/thermal cameras, light detection and ranging (LiDAR) and chemical sniffers, can be equipped to increase the functionality of an UAV. The wide variety of data collected can assist in the analysis of structural integrity [3,4].

Of the many different inspection methods used, an emerging method for UAV-based infrastructure monitoring is to generate 3D models using Structure from Motion (SfM). Ruggles et al. [5] explores UAV SfM and contests that SfM is an alternative to traditional LiDAR; Khaloo and Lattanzi [6] recognize that repeat inspections of infrastructure systems are needed and that SfM simplifies repeat inspections. SfM uses photographs to create 3D models of objects of interest. This is done by using multiple photos of an object from varying perspectives to determine spatial intersections [7]. Once the model is generated, it can be visually inspected or further analyzed using computer programs. Common types of post-processing analysis include surface-change mapping [8], anomaly detection [9], geologic structure mapping [10] and volume calculations [11]. SfM is popular because of its visual quality, portability, ease of data collection, and relatively low cost [12].

### 1.1. Camera Orientation Planning

View planning for SfM has been an active area of research for a variety of applications and is also sometimes referred to as camera orientation planning. As a general topic, view planning includes planning camera position and orientation to accomplish a given task. Huang and Savkin [13] mentions the importance 3D quality throughout models and point clouds in their view planning. In some cases, the planning includes specifying focal length or camera lens. This problem has been applied to a variety of vision tasks, some examples include surveillance [14], target tracking [15], quality control [16], coverage [17] and SfM [18].

For each of these problems the formulation typically is similar. Although some solutions have been formulated in continuous space [19], most view planning has been done using a theoretically countably infinite number of possible camera locations in discretized space. Liu summarizes typical view-planning problems as an input of models for the environment, cameras, and the required task [20]. The problem is discretized in space and a pool of possible camera position and orientations are generated. The camera orientation and terrain information is then used to generate a graph that describes what information each camera can capture. Several methods to generate this graph are described by Mavrinac and Chen [21]. The problem is formulated as the set covering problem and solved for the optimal camera placement strategy [22,23]. Recently, Shakhatreh et al. [24], who present a comprehensive literature review that includes addressing the challenge of remote sensing view planning, note that flight and view planning are still in the process of becoming optimized and repeatable.

### 1.2. Camera Orientation Planning for SfM

Several research groups study camera orientation planning (view planning) for SfM due to the computational complexity of processing large photo sets. Estimates from Bemis et al. [25] propose that doubling the number of photos will increase processing time for SfM models by four times and suggest that automatic UAVs can decrease required photo counts. There have been two approaches to overcoming this challenge. The first is to improve the SfM algorithms. There has been some success in this area, reducing portions of the problem to O(n), linear scaling with problem size, without sacrificing accuracy [26]; however, the reconstruction process is still $O(n^{2})$, quadratic scaling of computational cost with increasing problem size, limiting the speed at which SfM models are processed [27]. A second method to reduce the computational strain is to limit the number of photos needed for modeling through a more intelligent selection of camera position and orientation. This method applies general view-planning ideas to the SfM problem, reducing the processing load. In the current work, this is the approach that the authors undertake to address the computation constraints.

The general workflow as outlined by Liu et al. [20] applies to camera view planning for SfM modeling with some minor modifications to the traditional surveillance and coverage problems due to the unique requirements for model construction. The major modification centers around ensuring that each point is seen from multiple angles to ensure accurate reconstruction. Hoppe et al. solves the problem for range scanners by creating groupings based on the viewing angle relative to the surface normals [28]. This is later applied to SfM allowing for a regular view-planning formulation once the cameras are grouped [29]. On the other hand, Schmid et al. developed a view-planning algorithm that compares a potential candidate against already chosen cameras. This allows for finer control of the desired angles between cameras [30]; however, this method is relatively slow, making optimization computationally unfeasible for all but the smallest cases.

### 1.3. Iterative Modeling

In recent years there has been additional work on iterative modeling. Iterative modeling is to plan a high-level flight based on old or less accurate data and use the results from the first flight to plan a more precise flight based on the updated data. For example, Schmid et al. follows this process using a low resolution digital elevation model (DEM) for initial high-level flight planning and their updated surface model for subsequent refined flight planning [30].

In a similar study, Martin et al. explores iterative modeling as it applies to long linear features [31]. In their paper, they investigate the decoupled problem of a high-level inspection and low level (higher detailed) inspection of objects of interest discovered during the high-level flight. This unique approach allows for increased accuracy when objects of interest are discovered, while still maintaining coverage over the entire area for monitoring.

Infrastructure monitoring using 3D surfaces continues to evolve to address issues such as sharp features or the undersides of bridges. Recently iterative applications incorporated anomaly detection as in Morgenthal et al. [32] and using 3D point clouds (from previous flights) in Pan et al. [33] for additional analysis–both use bridges as case studies.

Iterative modeling allows for view planning with greater confidence without the need to apply probabilistic planning due to uncertainty. Although there has been significant research on planning the next best view in an unknown environment [34,35,36,37], for many regular inspection tasks, it is not necessary to add this additional level of complexity due to the availability of an already accurate model of the infrastructure. A simple high-level flight, or a prior model can be sufficient to precisely plan a subsequent flight. This also allows for greater confidence when planning flights around obstacles and in close proximity to the target surface.

### 1.4. Multi-Scale Modeling

A less studied area for SfM is the use of targeted multi-scale modeling. Although Martin et al. touch on the idea with their approach to monitoring long linear features, camera position and orientation for the two inspection passes are planned separately [31]. For regular inspections of infrastructure, there are often known areas of higher interest to the inspector due to the higher risk of failure. In some cases, thorough inspection of particular features is mandated by national inspection standards. In a recent publication, Khaloo et al. report a proof of concept study on multi-scale modeling [6] demonstrating that multi-scale photos can be used to achieve varied desired point densities for a model. Later the same group applies these findings to a test case in Khaloo et al. [38]. In this second study, the specified overlap and distances are used to create flight paths that are then flown manually. While they achieve desired accuracies, almost 5000 images are collected of the 85 m long bridge. The work in this paper bridges the gap between the work done by Khaloo et al. and the view-planning work already done for SfM.

Multi-scale expands on flight, view, and orientation planning which are stressed in Chiabrando et al. [39]. Bianco et al. [40] reviews the process of SfM and recognizes that multiple resolutions affect reconstruction; however, while a single multi-view stereo (MVS) pipeline is most consistent, the density of the point cloud is the key factor. Higher priorities produce denser point clouds and lower priorities are sparser, so it is assumed that if there is error introduced from matching across different scales that it is negligible. This assumption is based on the accurate dense point clouds produced from multi-scale SfM on a bridge by Khaloo et al. [38].

The studies by Khaloo et al. demonstrate the concept and the validation of using multi-scale infrastructure modeling [6,38]. This includes the use of point clouds with increased point density for finer inspection in select areas; however, as noted in the first paragraph of Section 1.4 Multi-Scale Modeling, the process did not include view planning or automated data acquisition. The results of the case study, in this paper, also yield 3D models from a single multi-scale inspection with tiered model quality that may be used for targeted inspection of infrastructure elements.

The work is validated on a test case. The three levels of detail in the case study correspond to the nature of the data required for achieving a feature-based inspection of infrastructure. In the test case of a dam, varied levels of precision are adequate for inspection of various elements of the dam. Accordingly, the multi-scale approach provides an automated optimized way to obtain the required information for accomplishing inspection tasks in a single 3D model with tiered quality according to known inspection needs. For example, the entire dam site requires general inspection, and elements such as the surrounding abutments, or the main embankments may be inspected with a limited level of detail. However, regions of the dam susceptible to certain failure modes (gradual or precursor to catastrophic), require a more precise inspection that a multi-scale approach enables.

### 1.5. Novel Contributions

The following novel contributions are included in this work:

- Demonstrate automated targeted infrastructure inspection using optimized multi-scale view planning for tiered model quality.
- Show consistent accuracy with tiered model quality based on priority level.
- Demonstrate the advantage of coupling view planning across multiple scales.
- Illustrate the potential resource efficiency of targeted non-homogeneous drone-based infrastructure inspection

## 2. Methods

**Figure 1:**
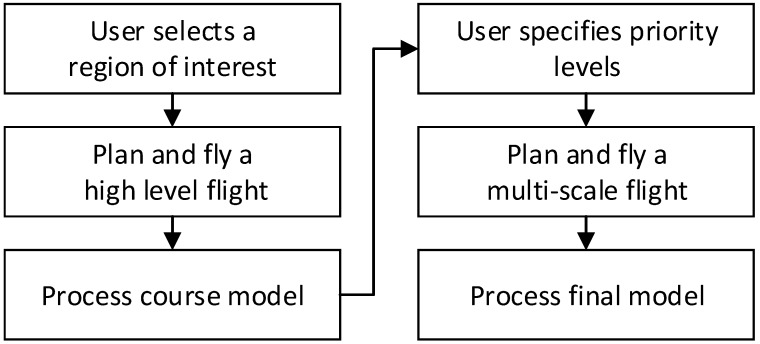
Overall workflow for iterative view planning.

The overall process for the 3D model creation in this study is outlined in Figure 1. For the multi-scale modeling, an initial coarse model is first obtained by selecting the inspection region, planning a high-level optimized flight and processing the photos using SfM. Once the base planning model is obtained, the user sets up the targeted inspection by choosing priority features and specifying a covering radius and a flight proximity for each priority level. The set covering problem is solved for that target and the generated camera position and orientation are stored and remembered when solving the set covering problem at different priority levels. Every set covering solution is combined to solve the TSP. The multi-scale inspection is then planned and flown, from which the final model is processed. Please note that for repeat inspections, a prior multi-scale model may be used in place of the coarse model, omitting the first three steps shown in Figure 1.

### 2.1. View-Planning Method

**Figure 2:**
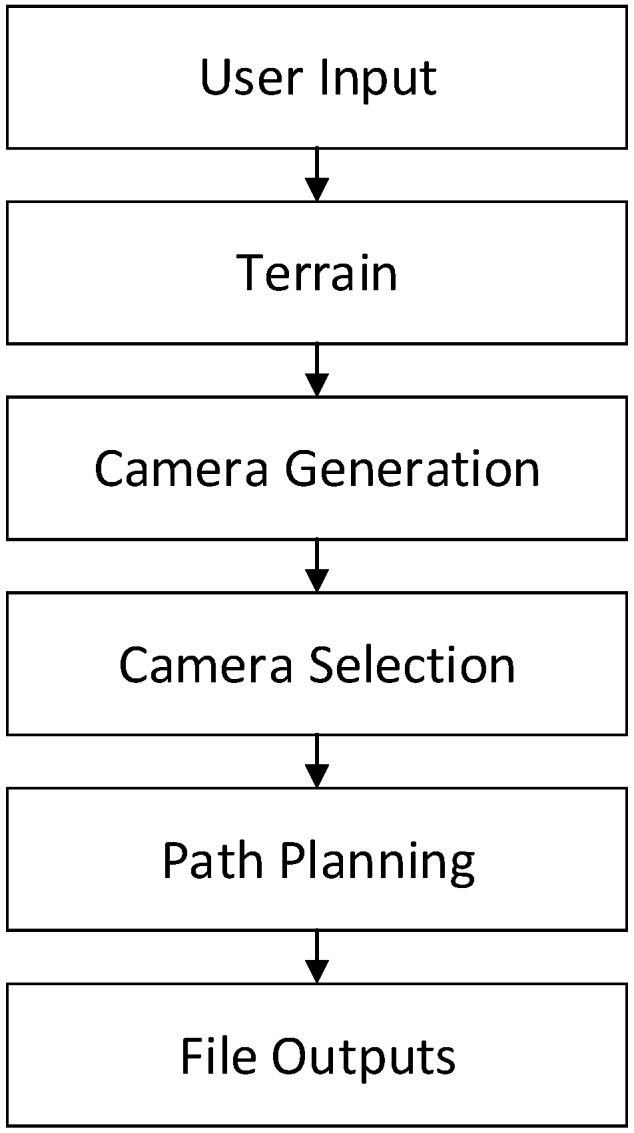
Overall workflow for camera orientation planning.

The view-planning method and basic problem formulation in this work is primarily built on the work presented in Martin et al. [31]. Each viewpoint is selected based on the a priori points, which can be seen, given the terrain and occlusions represented in the a priori model. [22] similarly ensure capture of features of interest, but [22] use geometric constructions to represent and plan the capture of the terrain. The general workflow of the current work is outlined in Figure 2, and key aspects are described in the following paragraphs.

The process begins with user input. For this step, the user first specifies spatial coordinates bounding the entire inspection region of interest and an imaging distance for general inspection (low priority—refer to Section 2.3 Site Selection for an application example). Next, the user specifies the spatial coordinates of target features or regions to be more closely inspected and the desired imaging distance for those inspection targets. The user then inputs camera information, including sensor aspect ratio and field of view, and flight information, including a minimum safety distance that the UAV must maintain away from objects. Finally, the user specifies the desired percent coverage (typically 90–95%) or the maximum number of images allowed.

The 90–95% coverage describes an a priori method to ensure effective tie points in 3D model reconstruction (not the expected coverage of the final model). From a histogram of possible camera locations and corresponding orientations, greedy heuristics select the camera that would capture the most a priori data points. A point is considered part of coverage once it has been predicted by 3 or more possible cameras. In terms of overlap, normally a nadir grid would need ≈70% image overlap for SfM; however, while this non-nadir coverage does not follow traditional overlapping metrics, the overlap is sufficient to create detailed reconstructions. This desired coverage is a user input.

With all the inputs from the user, the terrain information needed for view planning is prepared. Elevation data is obtained from the NED (National Elevation Dataset [41] as mentioned in Wang et al. [42]) for the area of interest using Matlab’s mapping toolbox [43]. If a previous point cloud is available for more detailed planning, it is used in conjunction with the NED. A triangle mesh of the terrain is then generated using the Delaunay triangulation method, included in the Python SciPy package [44]. Normals for each of the triangles are generated and stored for later use.

Next, potential camera position and orientations are generated. These are generated by placing a camera on each of the surface triangles’ normals at the user-specified distance from the surface. The cameras are oriented to look back down the normal, directly facing the surface triangle from which they are generated. The potential camera locations are then checked to ensure that they are not underground or within the user-specified safety distance from the surface. Cameras at invalid locations are removed from the camera set.

**Figure 3:**
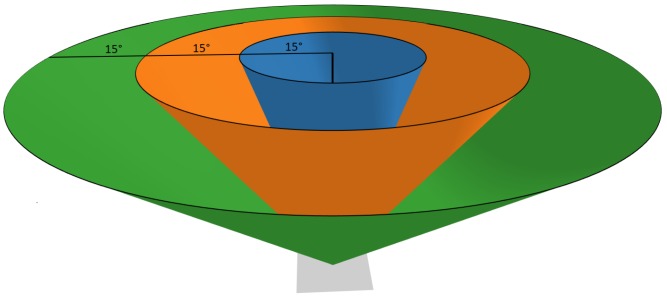
The different viewing angle for a single surface point.

Once the cameras are generated, an optimal camera set is selected. To do this, a graph of the cameras and which points they can see is generated. In addition to determining which cameras can see which points, the cameras are sorted by viewing angles as shown in Figure 3. If the angle is more oblique than 45°, the camera is marked as not viewing the point. This sorting of the buckets is the major difference between view planning for SfM and the more traditional view planning for coverage in surveillance applications. As stated previously, greedy heuristics are then used to select a subset of cameras that cover the user-specified region for each of the multiple viewing angle groups. Each potential camera is selected from a histogram of possible position and orientation. As the selection is greedy, the potential viewpoint that sees the most a priori points is chosen first. Remembering the a priori points which have been seen by the first camera, the viewpoint seeing the next greatest number of a priori points (to achieve desired point coverage for viewing angle groups) is chosen, and so on, until the desired point coverage is achieved by the set of viewpoints selected.

A minimum flight path is optimized for the selected set of camera position and orientations. This is done using the Christofides algorithm with a guaranteed solution within 1.5 times the global optimal value [45]. Due to the guaranteed quality of the solution, the TSP as presented in Christofides [45] is used, but recent research such as Genova and P. Williamson [46] and Bozejko et al. [47] expound possible improvements solving the TSP. If the camera set is large, the cameras are clustered by location and the Christofides algorithm is applied to each of the clusters. This has a minimal effect on the overall performance because battery limitations mean that UAVs are unable to collect the entire set of photos in a single flight. By pre-batching the photo groups, the path-planning computation time is greatly reduced without appreciably increasing flight time.

Finally, once everything is completed, an ordered list is created of the optimized camera position and orientations for the flight. These outputs are formatted to work with both in-house developed mobile flight applications as well as commercially available software.

The method described is used for the initial flight planning; however, for the multi-scale flight planning, several modifications are needed. First, as the elevation data used for the multi-scale flight is the previously built coarse SfM model, the previous flight is a safety precaution in the case that the flight occurs in a disaster zone with unknown obstacles; the multi-scale flight requires this information to fly the UAV closer, in a manner safe to people and equipment, that obtains higher resolution at points of interest. This SfM model is imported into the optimizer instead of data from the NED. For the multi-scale method, the camera selection optimization methods are performed serially for each of the priority levels, starting with the highest priority level. For each subsequent priority level, the portions seen by previously chosen camera sets are marked as seen. This prevents repeat information from being acquired in the coverage optimization for lower priority regions. Finally, once an optimal camera set is selected for each of the priority levels, the flight path is optimized to collect all the photos with the minimum distance traveled using the Christofides method [45].

### 2.2. UAV Platform

In the field validation for this work, the optimized flights are flown using the DJI Phantom 4 Pro. This platform is chosen for its relatively high accessibility and market penetration, with DJI controlling 72% of the worldwide drone market share [48]. The platform is selected to test the optimized flight due to ease of use and highly developed SDK. Key specifications for the platform’s camera are included in Table 1. The drone automatically flies with an in-house developed Android mobile application interfacing with DJI’s SDK. The image capture settings are set to auto mode during flights to collect photos from the pre-determined locations with associated gimbal poses (with pitch, yaw, and roll).

### 2.3. Site Selection

**Figure 4:**
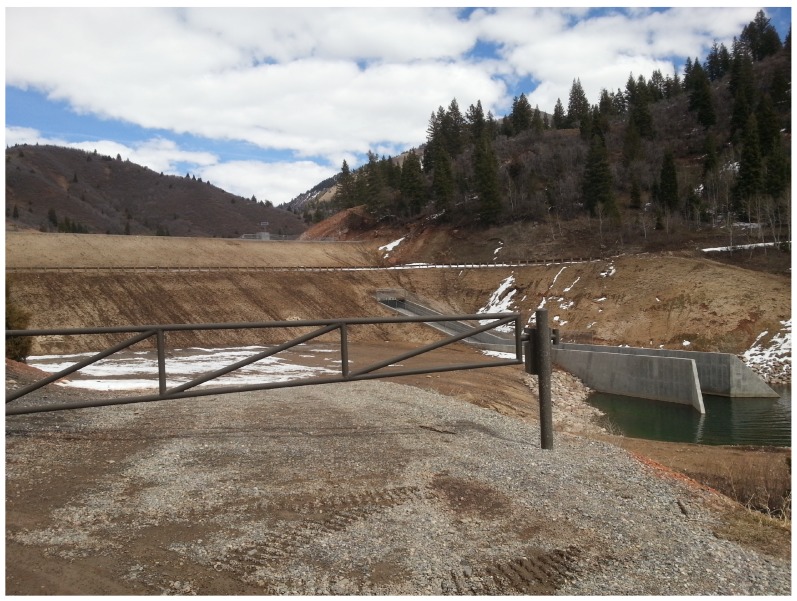
Tibble Fork Dam looking upstream.

The test site used for field validation in this study is Tibble Fork Dam, located in Utah, USA. Tibble Fork Dam is an earthen dam in the Wasatch Mountain Range approximately 260 m in crest length with a maximum stream to crest height of 25 m. The dam underwent a rehabilitation project during 2016–2017 to bring it into compliance with updated dam safety standards [49]. An image of the dam post-rehabilitation is shown in Figure 4.

**Figure 5:**
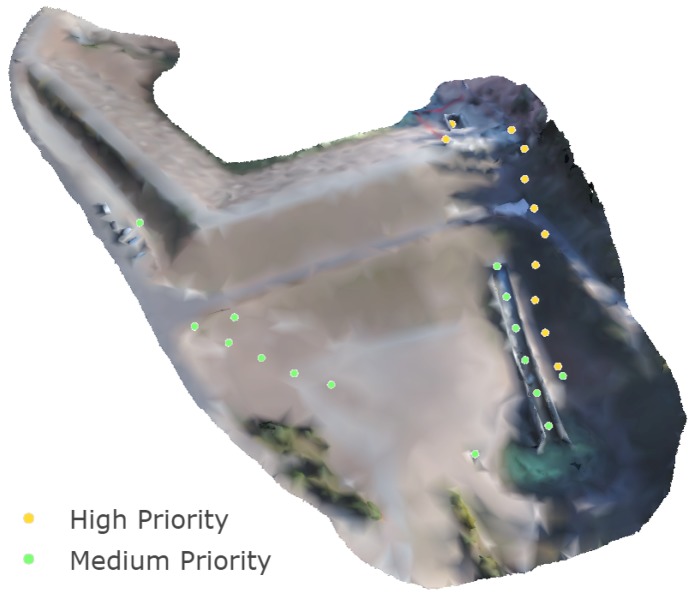
Coarse down-sampled model used for multi-scale modeling with priority points indicated.

The dam was selected as the practical validation site for this work because of its need for regular, comprehensive inspection. A typical inspection routine for an earthen dam such as Tibble Fork Dam includes careful visual inspection of structural health and identification of hazardous anomalies such as seepage exits, excessive surface weathering and erosion, deterioration of structural features, and vandalism. Current practice for inspection of earthen dams involves operations and maintenance personnel walking up and down the groins of the dam (the contacts between the embankment and the abutments), alongside the spillway structure, around the control house and spillway intake, and around other appurtenant features such as toe drain outlets or downstream weir structures. Other regions, such as the central embankment, are generally viewed from a distance, with less rigorous examination. According to these inspection tasks, high- and medium-priority features are selected for the multi-scale targeted view-planning method. Figure 5 shows the location of priority points on the dam. A 7 m radius buffer is applied around priority points to form priority regions. All regions on the dam not assigned a high or medium priority level are designated as low priority for inspection planning. Flight proximities of 15, 30, and 60 m are chosen for the high, medium, and low priority levels, respectively.

### 2.4. Establishing Ground Control and Validating Model Accuracy

**Figure 6:**
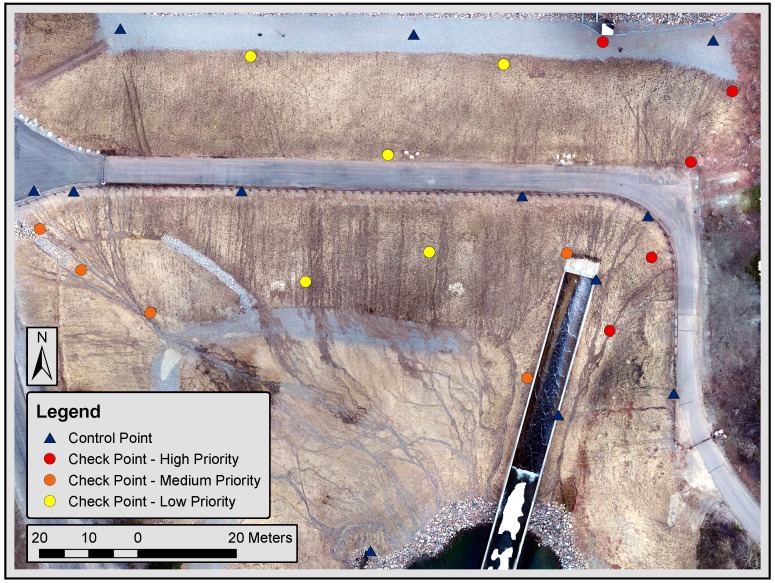
Control and check point locations on the face of Tibble Fork Dam.

Establishing ground control and validating SfM model accuracy are performed by collecting high precision ground survey data and comparing processed SfM 3D models to surveyed ground control. Twelve static features on the dam structure are chosen as ground control points (GCPs) for providing spatial referencing to the SfM model. An additional 15 targets are placed on the dam surface to use as check points (CPs) for model accuracy, with five points for each of the three priority levels. The 15 CPs are not used to spatially reference the SfM model but are used only to measure model accuracy at sample locations. Figure 6 shows GCP and CP locations. All points are surveyed by a Trimble S8 robotic total station and TSC3 data collector from two setup locations at the dam. The mean three-dimensional difference between the two surveys is 1.2 cm. The average between these two coordinate datasets is used in the accuracy analysis.

### 2.5. SfM Model Processing and Analysis

The SfM model processing is performed using Agisoft PhotoScan Professional [50]. Processing of the SfM model consists of Agisoft’s standard workflow: alignment of photos to create a sparse cloud, manual identification of GCPs and CPs in images, optimization of camera alignment, generation of a dense point cloud and generation of a 3D textured mesh. Default PhotoScan values are used for processing with a couple of the key settings listed in Table 2. PhotoScan is selected as the primary modeling program based on performance, ubiquity in the literature [51,52,53] and processing reports. Eventually, the density of the point clouds, the error in location of the GCPs and CPs, and comparisons of time and photos required demonstrates the merit of multi-scale models and methods. Visual comparisons are also made using Bentley ContextCapture [54] because of meshing ability and ease of model navigation [55].

The processed multi-scale SfM model is comparatively analyzed by priority region for accuracy, resolution, visual clarity, and reconstruction quality. GCP and CP error residuals in the SfM model are analyzed using JMP^®^ [56]. Resolution mapping is performed in CloudCompare [57] (which is open source) and visual clarity and reconstruction quality are assessed via Acute 3D Viewer [54].

## 3. Results and Discussion

Results of the test case at Tibble Fork Dam are presented below, including flight planning and SfM modeling results, as well as model quality analysis based on spatial accuracy, resolution, visual clarity, and reconstruction quality.

### 3.1. Flight Planning

**Figure 7:**
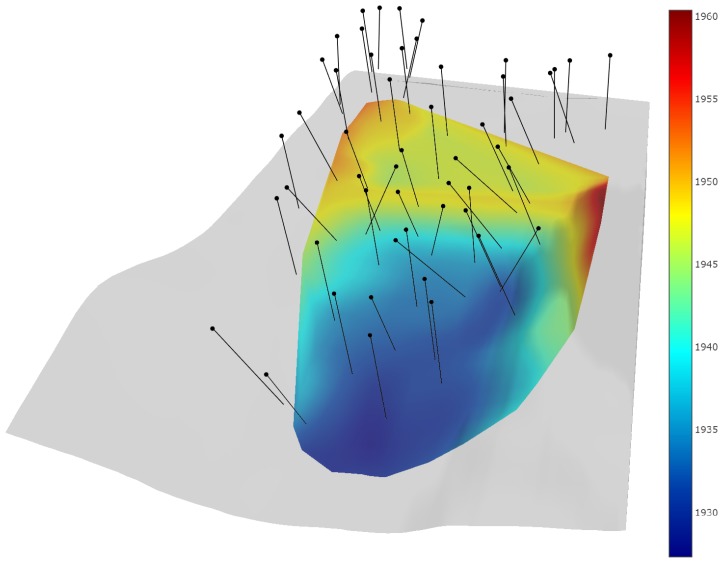
Initially optimized camera location and orientations are based on publicly available elevation data. The initial optimized flight ensures safety to people and equipment while recreating a quicker fuller model than a grid flight. Elevation units are m.

The initial optimized flight is planned at 80 m using the publicly available NED terrain data (see Figure 7). In total, there are 51 optimized photo locations. The flight required 11 min to fly using the DJI phantom 4. Following the flight, model processing in Agisoft PhotoScan required a total of 3 h and 35 min. The processed model of Tibble Fork Dam was down-sampled from 3.3 million faces to 10 thousand faces for planning the multi-scale flights. Considering the significant down-sampling performed, the coarse model could be processed at a reduced quality to save processing time.

**Figure 8:**
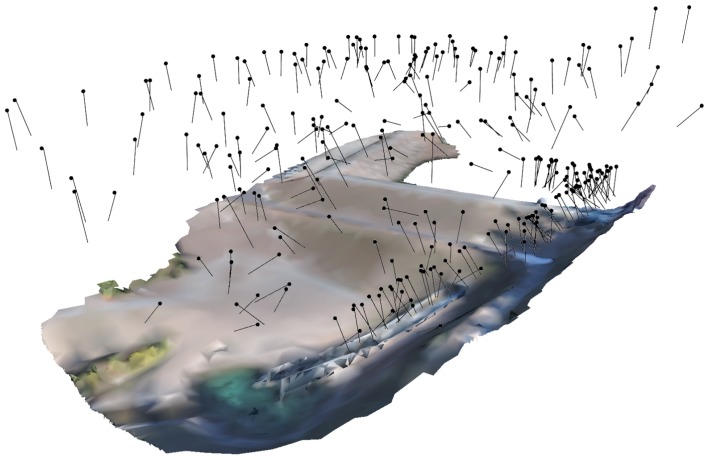
Final optimized camera position and orientations for the multi-scale flight.

The optimized multi-scale flight had a total of 266 camera locations (see Figure 8) and took a total of 350 s to run on an Intel Core i7-3630QM processor with a clock speed of 2.4 GHz and 8 GB of RAM. The breakdown for the number of cameras and solve time by priority region is included in Table 3. The high-priority region requires a relatively large number of cameras when compared to the medium priority region. This difference is due to the significant overlap between the high and medium sections on the left side of the dam and the comparatively small field of view at high-priority distance from the surface. The low priority region requires a large number of cameras due to significantly greater surface area.

To assess the value of multi-scale inspection, single-scale optimized flights for the entire dam are planned at each of the priority levels. The results and computational time for these flight plans are included in Table 4. As is expected, the higher the priority (or the closer the flight) the more photos needed to adequately model the entire dam. If the entire dam were flown at 15 m, over 1000 photos would be needed. By using a multi-scale tiered system, the number of photos needed for the inspection of the dam is reduced by 75%. This reduces the resources required for field inspection and translates to reduced processing time when constructing a model from the photos [27]. When comparing Table 3 and Table 4, again note that serial camera selection in the multi-scale method avoids repeat coverage at lower priority. For example, low priority inspection of the entire site requires 159 photos; however, for the multi-scale flight, higher priority photos cover a portion of the site, reducing the total number of needed photos for low priority inspection to 141. While this detail may seem expected, to the authors’ knowledge, it has not previously been demonstrated in multi-scale SfM view planning.

The dam was flown by the DJI phantom 4 Pro in three flights of approximately 90 photos each. Each flight took 17 min to collect the 90 photos. Including the time between flights, the entire photo collection period took 1 h.

### 3.2. SfM Modeling

**Figure 9:**
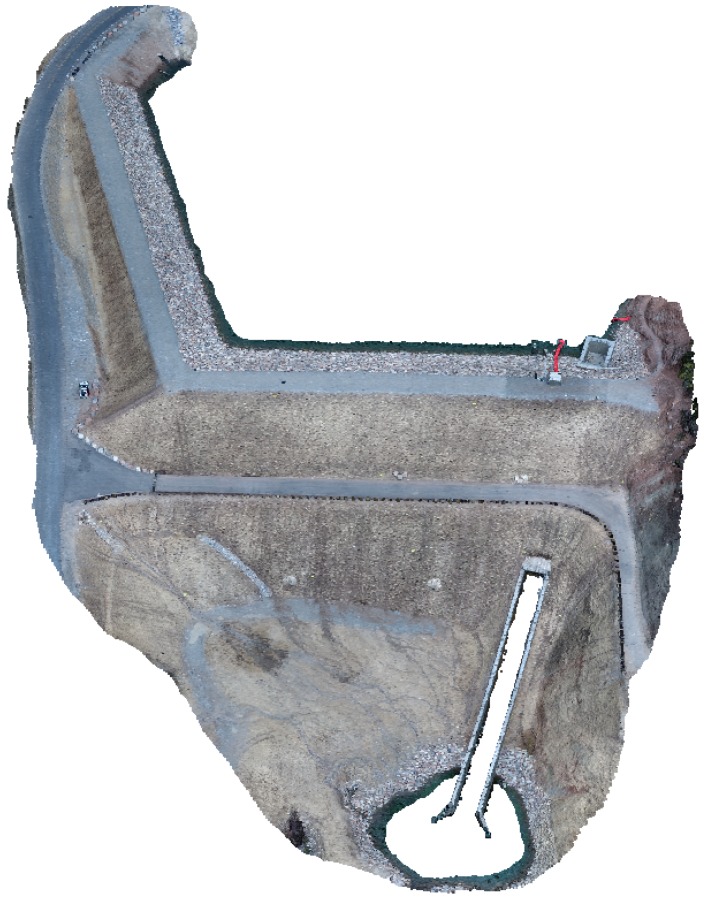
Final model of Tibble Fork Dam built using Agisoft PhotoScan software.

SfM model processing results are shown in Table 5, and the finished 3D model is displayed in Figure 9. The 266 images (each 18 megapixels) processed in Agisoft PhotoScan generated a dense cloud with 132 million points and a 3D model with 26 million faces. Though the model resolution is non-homogeneous, as discussed in a subsequent section, the average ground surface resolution (also commonly referred to as ground sampling distance or GSD) is 9.4 mm/pixel. The model was processed on an Intel(R) Xeon(R) E5-2680 v2 machine with a clock speed of 2.80 GHz, 256 GB RAM, and an NVIDIA GeForce GTX 1080 GPU. Processing time for each step is shown in Table 5.

### 3.3. Model Accuracy

Each of the CPs in the final model are compared to the respective total station measurement to compute the error for the CPs in each of the regions. The average error for each of the regions (including the respective 95% confidence intervals (CI)) are given in Table 6. The results follow the expected trend of increasing accuracy with increasing priority; however, the 95% CI overlap with the other priority levels, indicating that there is statistical overlap in accuracy between each of the regions.

A Tukey-Kramer comparison of multiple means is used to compare the priority regions to one another. This method with pooled standard deviation is appropriate because the standard deviations for each of the priority regions are similar (0.97, 0.35 and 0.43 cm for the low, medium, and high-priority regions, respectively). The results from this test are shown in Table 7. The reported CI are for the 95% confidence region. In all cases the p-value for the null hypothesis (that there is no difference between the regions) is much greater than the 0.05 cutoff; therefore, there is no evidence that any of the methods has a statistical difference between it and another method. One possible reason for the lack of evidence that the methods are different is the relatively small sample size used. Assuming that the estimated standard deviation and the calculated means are correct, to have no overlap in the CI, a total of 22 points for each priority region would be needed. Additionally, as mentioned in Section 2.4, the average variation between the two repeat field surveys is 1.2 cm. This inherent error in the measurements is within the 95% interval, indicating that much of the calculated SfM model error could be due to the error in the measurement accuracies with the Trimble S8 robotic total station. To mitigate this error, the average of the two total station surveys is used. Another important factor influencing model error and the limit to which differences in model accuracy across priority levels can be detected is the manual identification of control and CPs in images as part of the SfM modeling process. In performing manual identification of control and CPs, it is generally easier to precisely define ground survey points in higher priority images because of the finer resolution, but even at high-priority level, limited image resolution introduces potential error in point selection on the order of 0.5 cm. While there is no statistically significant difference in model accuracy across priority levels, mean accuracy for the three priority levels follows the expected trend of higher accuracy at higher priority level, and the overall mean SfM model accuracy is 1.3 cm.

### 3.4. Model Resolution

**Figure 10:**
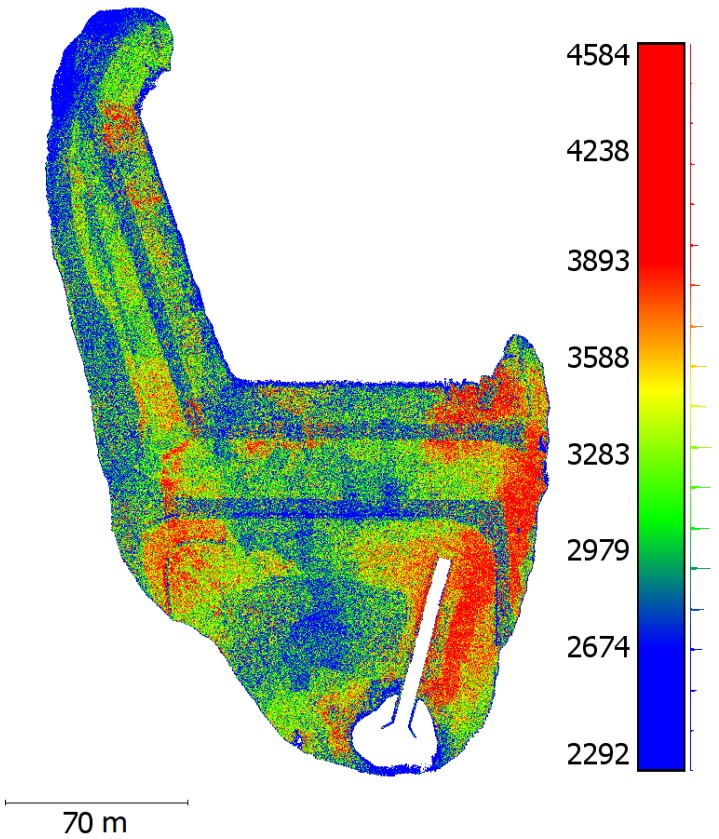
A map of the resolutions calculated as the number of surface points within a 1 cm radius.

The multi-scale view planning image acquisition resulted in an SfM model with tiered model resolution. Figure 10 shows a map of the dam with the scalebar indicating surface point densities per 1 cm radius. While there is some noise and scatter in the resolution map, examining Figure 10 alongside Figure 5 shows that the achieved resolution aligns quite well with the selected priority regions for inspection planning. Using mid-range point densities of 2800, 3500 and 4100 points per unit area from Figure 10, resolution for medium and high-priority regions are approximately 25 and 50% higher, respectively, compared to low priority regions. As point density derives directly from pixel density, this highlights how the multi-scale planning approach concentrates SfM processing load where high-resolution data is most needed for inspection. Achieving higher resolution modeling of critical structures in priority regions also has the potential to facilitate computer vision-based anomaly detection in post-processing model analysis, such as the work done in [9].

### 3.5. Visual Clarity

**Figure 11:**
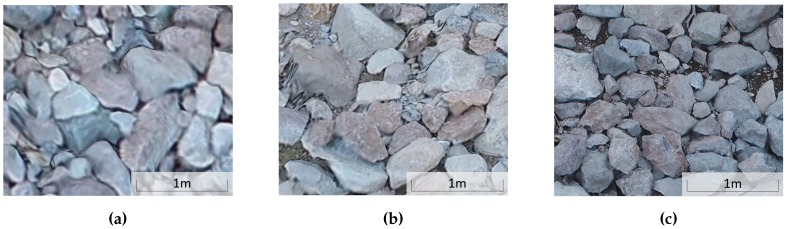
Visual comparison of riprap extracts from (**a**) low, (**b**) medium and (**c**) high-priority regions of final SfM model.

Inspection of the multi-scale model shows that visual clarity improves significantly by priority level. Figure 11 displays samples of modeled riprap at each priority level. The medium and high-priority regions have much finer detail in the model than the low priority region. Though such a simple visual comparison of model quality may seem trivial, the end use of SfM models is often visual qualitative evaluation of infrastructure. In this case, model clarity in needed areas is highly valuable. While an ideal model might have excellent visual clarity in all locations, targeted collection better meets inspection objectives while limiting collection and processing costs, as discussed at the beginning of Section 3.1. Results of this study have shown that tiered visual clarity is obtained in an automated inspection, achieving higher visual clarity where needed and lower visual clarity where higher clarity is less needed.

### 3.6. Reconstruction Quality

**Figure 12:**
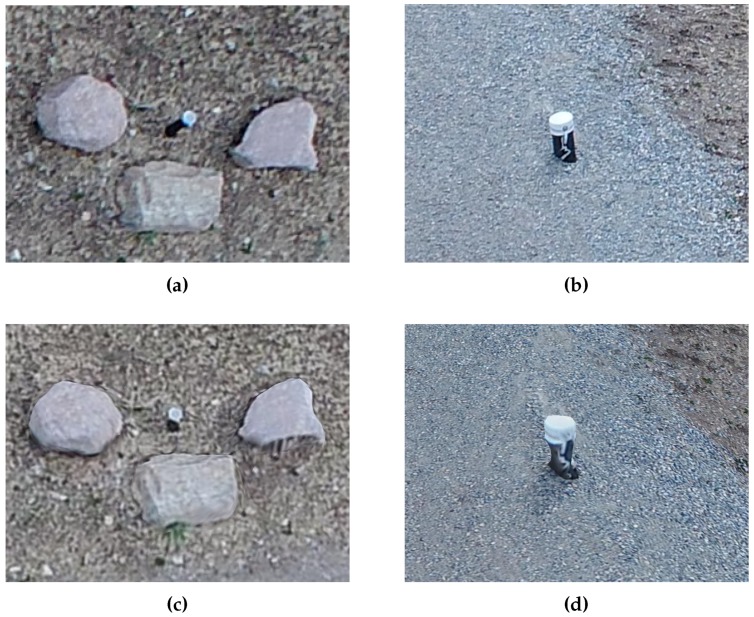
Visual comparisons of instrumentation features in (**c**) low and (**d**) high-priority regions. Corresponding photos are included in (**a**,**b**). Peizometer pipe in (**a**,**c**) is 11 cm in diameter and protrudes approximately 0.55 m from ground surface. Spillway drain access pipe in (**b**,**d**) is 17 cm in diameter and protrudes approximately 0.36 m from ground surface.

**Figure 13:**
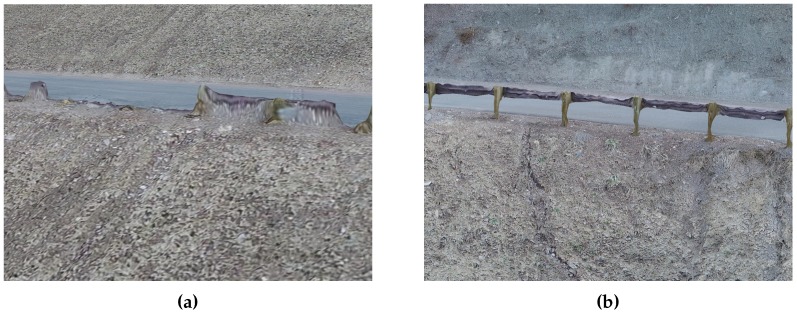
Visual comparison of guardrails in the (**a**) low and (**b**) high-priority regions. Guardrail spacing is 1.9 m.

Analysis of the SfM model showed tiered reconstruction quality by priority region. Higher priority regions have reduced flattening effects on protruding features, reduced warping of angular features and better reconstruction of open features. SfM programs generally have difficulty accurately reconstructing angular, protruding and open (non-solid) features. Often, such features are warped, non-existent, or flattened in SfM models. As expected, image sets containing ample close views of varying perspective and obliquity improve the reconstructability of such features. Analysis of the multi-scale model shows a step improvement in reconstruction of features difficult for SfM. Figure 11 shows reduced warping and spanning of angular riprap. Figure 12 shows successful reconstruction of a protruding spillway drain access pipe in high-priority region compared to pixels of a piezometer pipe flattened onto the ground surface in low priority region. Finally, Figure 13 shows more successful reconstruction of “open” guardrails in high priority compared to low priority. These examples demonstrate the improved quality of SfM model reconstruction with increasing priority level, again validating the achievement of tiered model quality through multi-scale view planning.

## 4. Conclusions and Future Work

This paper demonstrates automated targeted infrastructure inspection using optimized multi-scale camera view planning for tiered model quality. The work is validated at Tibble Fork Dam in Utah. As the dam structure has recently been modified, the site was flown iteratively with a first flight to generate a more accurate surface model for view planning. Camera locations for targeted multi-scale SfM modeling were optimized and the mission was automatically flown using a multi-rotor UAV with a gimballed camera. Models were processed using Agisoft PhotoScan and analyzed across priority levels for accuracy, resolution, visual clarity, and reconstruction quality. As measured against total station surveyed CPs, there is no statistically significant difference in the model accuracy across priority levels, with a mean model accuracy of 1.3 cm. Model resolution is approximately 25 and 50% higher for the medium and high-priority regions, respectively, when compared to the low priority region. Visual inspection of the SfM model shows marked improvement by priority level, with higher priority regions capturing more detail. Reconstruction of features difficult for SfM modeling is qualitatively superior at higher priority levels. These findings validate the use of optimized multi-scale view planning to accomplish automated targeted infrastructure inspection with tiered SfM model quality. Future work includes adapting this multi-scale targeted optimization framework to multi-sensor inspections, change detection with targeted inspection and automatic feature detection with machine learning to inform the multi-scale targeted regions.

Greedy heuristics are used to optimize the location of each camera. A complete analysis of different optimization methods against the performance of the Greedy algorithm will help identify time-memory trade-offs. This analysis will aid in implementing multi-scale planning in real time. Modifications to the optimized view-planning algorithm that specifically prevent rotations around the projection center could expand the multi-scale method to more complex 3D shapes. Additionally, the method described in this paper relies on the availability of elevation data or a previous point cloud model. The modifications required to use a multi-scale targeted approach in an unknown environment are yet to be explored.

## Figures and Tables

**Table 1:** Basic camera specifications for the DJI Phantom 4 Pro.

Sensor	1″ CMOS
Field of View	77.7°
Image Size (Pixels)	4864 × 3648
Horizontal Viewing Angle	62.1°
Vertical Viewing Angle	46.6°
3-Axis Gimbal	−90° to +30°

**Table 2:** Several key model processing parameters used in Agisoft PhotoScan for model processing.

Alignment accuracy	Highest
Alignment key point limit	40,000
Alignment tie point limit	4000
Reconstruction quality	High
Reconstruction depth filtering	Aggressive

**Table 3:** The number of cameras and the computational timing results for each of the optimized sections.

	High	Medium	Low	Total
Distance (m)	15	30	60	
Time (s)	7.7	11.1	331.6	350.4
Cameras Selected	99	26	141	266

**Table 4:** Computational time and number of photos needed to model the entire Tibble Fork Dam at varying priority levels.

	High	Medium	Low	Multi
Distance (m)	15	30	60	15–60
Time (s)	1344	674	372	350.4
Cameras Selected	1063	434	159	266

**Table 5:** PhotoScan processing details for final model generation.

Processing Phase	Points/Faces	Time (h)
Alignment & Sparse Cloud	261,139	0.18
Dense Cloud	132,428,026	2.57
3D Textured Model	26,485,581	4.00
Total		6.74

**Table 6:** The average error in each of the priority regions for the CPs. All units are in cm.

Level	Mean	Std Error	Lower 95%	Upper 95%
Low	1.62	0.29	0.99	2.24
Medium	1.25	0.29	0.63	1.88
High	1.00	0.29	0.37	1.63

**Table 7:** Tukey–Kraimer comparison of multiple means. All units in cm.

Level	−Level	Difference	Std Err Dif	Lower CI	Upper CI	*p*-Value
Low	High	0.61	0.41	−0.47	1.70	0.32
Low	Medium	0.36	0.41	−0.73	1.45	0.66
Medium	High	0.25	0.41	−0.83	1.34	0.81
